# Physiological Responses of *Rosa rubiginosa* to Saline Environment

**DOI:** 10.1007/s11270-017-3263-2

**Published:** 2017-01-28

**Authors:** Tomasz Hura, Bożena Szewczyk-Taranek, Katarzyna Hura, Krzysztof Nowak, Bożena Pawłowska

**Affiliations:** 1 Polish Academy of Sciences, The Franciszek Górski Institute of Plant Physiology, Niezapominajek 21, 30-239 Kraków, Poland; 2 Department of Ornamental Plants, Faculty of Biotechnology and Horticulture, University of Agriculture in Kraków, Al. 29 Listopada 54, 31-425 Kraków, Poland; 3 Department of Plant Physiology, Faculty of Agriculture and Economics, University of Agriculture in Kraków, Podłużna 3, 30-239 Kraków, Poland; 4 Department of Dendrology and Landscape Architecture, Faculty of Biotechnology and Horticulture, University of Agriculture in Kraków, Al. 29 Listopada 54, 31-425 Kraków, Poland

**Keywords:** Salinity, Chlorosis, Chlorophyll fluorescence, Photosynthesis, Leaf anatomy

## Abstract

The aim of this work was to analyse the response of *Rosa rubiginosa* to salinity induced by different concentrations of sodium chloride and calcium chloride (0, 25, 50, 100, 150 and 200 mM). Besides salt accumulation and pH changes, other parameters were investigated including photosynthetic activity, leaf water content, the dynamics of necrosis and chlorosis appearance and leaf drying. The study was complemented with microscopic analysis of changes in leaf anatomy. *R. rubiginosa* was more sensitive to the salinity induced by calcium chloride than by sodium chloride. Plant response to salinity differed depending of the salt concentration. These differences were manifested by higher dynamics of necrosis and chlorosis appearance and leaf drying. CaCl_2_ showed greater inhibition of the photosynthetic apparatus and photosynthetic activity. Treatment with CaCl_2_ caused more visible deformation of palisade cells, reduction in their density and overall reduction in leaf thickness. The study demonstrated higher accumulation of CaCl_2_ in the soil, and thus greater limitations in water availability resulting in reduced leaf water content and quicker drying of leaves as compared with NaCl-treated plants.

**Electronic supplementary material:**

The online version of this article (doi:10.1007/s11270-017-3263-2) contains supplementary material, which is available to authorized users.

## Introduction

**Fig. 1:**
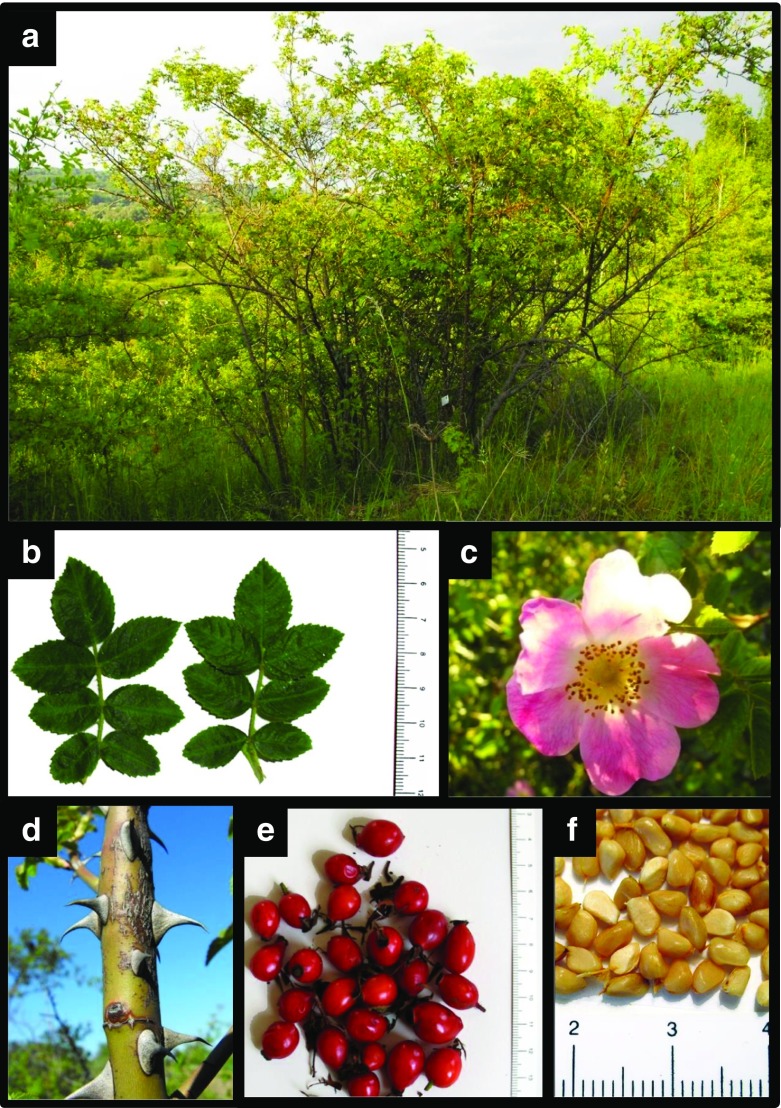
*R. rubiginosa* in its natural environment in the southern Poland (Małopolska, near Kraków) (**a**), leaves (**b**); flower (**c**); shoot with prickles (**d**); fruit (**e**); seeds (**f**)

*Rosa rubiginosa* is native to the entire area of Europe (Zimmermann et al. 2010, 2011). It is a fast growing shrub of bushy and branching habit (Fig. 1). It prefers sunny spots but grows well in slightly semi-shaded places. The species is tolerant of soil drought, urban contaminants, poor soils, frost and diseases (Kissell et al. 1987; Monder 2004a, b, 2012; Ritz et al. 2005; Sage et al. 2009; Svriz et al. 2013). Its resistance to adverse environmental conditions makes *R. rubiginosa* widely useful in urban green areas of natural character and in reclamation of polluted urban soils (De Pietri 1992; Sheley et al. 1996).

Many years of anthropogenic activity in urban areas caused considerable changes in the morphological, biological, physical and chemical properties of urban soils and destruction of their structure and nature (Pouyat et al. 1995). The soils of green strips surrounding urban communication routes contain also high concentration of salt (NaCl, CaCl_2_) as a consequence of using chemicals to make the roads less slippery (Cunningham et al. 2008; Novotny and Stefan 2010; Zeng et al. 2012). Chemical agents react with ice and water which do not freeze (the freezing point of water is reduced by the salt). Then, chlorides from melting snow get into the soil changing its chemical composition. A common effect of this approach is the death of roadside trees and shrubs due to physiological drought or disturbances in nutrient absorption (Czerniawska-Kusza et al. 2004; Di Tommaso 2004).

Considering its high effectiveness in soil reclamation, resistance to environmental stresses and positive influence on other plants, *R. rubiginosa* seems a useful species to be planted on salt-contaminated urban soils along the communication routes (Williams 1997).

The rate of metabolic disturbances and adaptability processes under salt stress differ depending on halophytic or glycophytic properties of species (Bankaji et al. 2016; Bowman et al. 2006; El-Haddad and Noaman 2001; Han et al. 2012; Redondo-Gómez et al. 2009). In the soils containing high concentrations of salt, water absorption by plant roots is limited (Zhang et al. 2016). This results in physiological drought manifested by a decrease in cell water content, stomatal closure and reduced photosynthetic performance (Nandy et al. 2007; Zhang et al. 2010). Therefore, an assessment of salt stress effects on the activity of photosynthetic apparatus in *R. rubiginosa* may be crucial to determine the level of salt tolerance of this species.

The aim of this study was to evaluate *R. rubiginosa* response to the salinity caused by sodium chloride and calcium chloride. *R. rubiginosa* may be a natural indicator of salt contamination in the soils surrounding the urban roads, and its presence may facilitate the assessment of an ecological status of such areas and their vegetation. Our research hypothesis assumed that variable salt concentrations would induce different response of *R. rubiginosa*. We analysed the activity of its photosynthetic apparatus, dynamics of necrosis and chlorosis appearance and drying of the leaves. The study was complemented by microscopic analysis of changes in the leaf anatomical structure.

## Materials and Methods

### Plant Material, Growth Conditions and Treatments

The current study was performed in young *R. rubiginosa* plants. The seeds used in the experiment (Fig. 1f) were collected at the turn of October and November from a shrub growing in natural conditions in the southern Poland (Fig. 1a). They were sown following a standard warm and cold stratification, 10 weeks at 25 °C and 13 weeks at 3 °C. The seedlings were grown in a greenhouse (at day/night temperature of 25/20 °C ± 2 °C; photosynthetic photon flux density, PPFD, from 150 to 200 μmol (photons) m^−2^ s^−1^), in 9 cm diameter pots filled with Klasmann-Deilmann TS1 substrate. During growth and before flowering, the plants were chemically protected against powdery mildew and downy mildew. The experimental plants were cut at 15–20 cm and each of them had about 14 healthy leaves.

They were treated with NaCl and CaCl_2_ solutions at 0 (control), 25, 50, 100, 150 and 200 mM. The plants were treated with salt solutions for 32 days. pH of the substrate prior to the experiment was 6.8, and electrical conductivity (EC) of the soil solution was about 300 μS (Elmetron CPC-401, Zabrze, Poland). EC and pH of the substrate were analysed after 14 and 32 days of salt treatments. EC and pH values are average of three replicates.

### Measurements and Analyses

During the experiment, the processes of necrosis, chlorosis and leaf drying were observed, and leaves showing evident these visible symptoms were counted on plant and expressed as percentage of control. Assessments of necrosis, chlorosis and leaf drying were taken in three replicates. A replication in an experiment represents a single plant (e.g. three replicates means three plants).

Leaf photosynthetic activity was assessed with Plant Vital R 5030 device (INNO-Concept GmbH, Germany). The molecular oxygen released from leaf surface of *R. rubiginosa* during photosynthesis under red light (*λ* = 630–650 nm) was measured directly by means of Clark electrode. The Clark-type electrode enables to detect trace amounts of oxygen produced by PSII. The measurement temperature was maintained at a constant level. Photosynthetic activity was defined based on the amount of oxygen released from specific leaf area (1 m^2^) within specific time (1 min). The following measurable parameters were estimated: *R* (mg/l · s) — oxygen evolving activity rate during the dark phase and *S* (mg/l · s) — oxygen evolving activity rate during the light phase between the minimum and maximum points. These two parameters were used to calculate the photosynthetic activity coefficient *K*_phA_ = −*S*/*R*. The measurements were taken in five replicates.

Chlorophyll fluorescence was measured with a fluorometer Mini-PAM (Walz, Effeltrich, Germany). To estimate maximum photochemical efficiency (*F*_v_/*F*_m_), the leaves were adapted to darkness for 20 min. *F*_v_/*F*_m_ was calculated according to van Kooten and Snel (1990) as (*F*_m_ − *F*_0_)/*F*_m_, where *F*_0_ and *F*_m_ represented the minimum and maximum chlorophyll fluorescence, respectively. The minimum fluorescence was determined by switching on the modulated red light (600 nm). The maximum fluorescence with all PSII reaction centres closed was determined by a 0.7 s saturating pulse at 8000 μmol m^−2^ s^−1^ in dark-adapted leaves. The measurements were taken in five replicates.

Leaf water content (LWC) and leaf dry weight (LDW) were measured by quantitative sampling of leaf fresh weight (*L*_FW_), followed by drying at 80 °C for 24 h. The resulting leaf dry weight (*L*_DW_) was assessed and water content was calculated according to the following equation and expressed as a percentage: LWC = ((*L*_FW_ − *L*_DW_)/*L*_FW_) · 100%. The measurements for LWC and LDW were taken in seven replicates.

Leaf anatomy was observed and photographed using a light microscope Jenaval (Carl Zeiss, Jena, Germany). A terminal leaflet of a compound leaf was harvested on the 12th day of the experiment. The plant material was fixed in glutaraldehyde (Sigma-Aldrich) (Forssmann 1969) and rinsed in 0.1 M phosphate buffer. Then, the leaf samples were dehydrated in ethanol and acetone and embedded in Epon 812 (Sigma-Aldrich) (Luft 1961). Resin blocks were cut with Tesla 490A ultramicrotome (Brno, Czech Republic). One micrometre thick sections were stained with Azure II (Sigma-Aldrich) and toluidine blue (Sigma-Aldrich) (Richardson et al. 1960).

## Results

### pH and Soil Salinity

Table 1 presents changes in soil pH and salinity depending on the concentration of NaCl and CaCl_2_ solutions used for watering of *R. rubiginosa*. After 14 days of treating with sodium chloride, the soil pH was neutral (pH = 7.0) or slightly alkaline (pH = 7.1–7.3) and after 32 days it was either slightly alkaline (pH = 7.0) or slightly acidic (pH = 6.7–6.9). The soil treated with CaCl_2_ was slightly acidic after both 14 (pH = 6.3–6.6) and 32 days (pH = 6.1–6.4) (except for 0 mM variant).

**Table 1:** pH and electrical conductivity (EC) of the soil after 14 and 32 days of treating *R. rubiginosa* with NaCl and CaCl_2_ of different concentrations (0, 25, 50, 100, 150, 200 mM). Mean value (*n* = 3) ± SE

	pH		EC [mS]	
	14 days	32 days	14 days	32 days
NaCl (mM)				
0	6.8 ± 0.13	7.1 ± 0.08	0.3 ± 0.08	0.4 ± 0.06
25	7.3 ± 0.10	7.1 ± 0.09	1.4 ± 0.32	3.7 ± 0.63
50	7.1 ± 0.05	6.9 ± 0.06	2.8 ± 0.20	7.7 ± 0.68
100	7.0 ± 0.06	6.7 ± 0.07	6.0 ± 0.07	12.5 ± 0.65
150	7.2 ± 0.09	6.9 ± 0.06	6.4 ± 0.20	16.4 ± 0.54
200	7.3 ± 0.04	7.1 ± 0.07	7.0 ± 0.15	18.0 ± 0.44
CaCl_2_ (mM)				
0	7.0 ± 0.02	7.1 ± 0.07	0.3 ± 0.03	0.6 ± 0.10
25	6.6 ± 0.09	6.4 ± 0.08	1.1 ± 0.08	6.5 ± 0.69
50	6.6 ± 0.03	6.2 ± 0.06	4.9 ± 0.09	8.4 ± 0.85
100	6.4 ± 0.05	6.2 ± 0.04	6.6 ± 0.71	14.9 ± 0.79
150	6.4 ± 0.04	6.1 ± 0.03	9.7 ± 0.26	18.5 ± 0.80
200	6.3 ± 0.04	6.2 ± 0.06	12.9 ± 0.19	20.6 ± 0.30

Conductometric measurements of soil salinity showed higher salt content for specific concentrations (except for 25 mM after 14 days) in CaCl_2_ variant than in NaCl one. After 14 days, the salt content for the highest concentration of 200 mM was about two times higher for CaCl_2_ than for NaCl.

### Assessment of Chlorosis, Necrosis and Leaf Drying

**Fig. 2:**
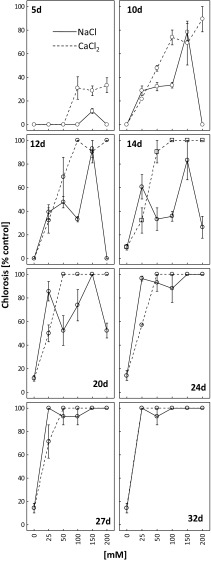
Dynamics of chlorosis appearance and its intensity as percent of the control after 5, 10, 12, 14, 20, 24, 27 and 32 days of treating *R. rubiginosa* with NaCl (*solid line*) and CaCl_2_ (*dashed line*) solutions at various concentrations (0, 25, 50, 100, 150, 200 mM). Mean value (*n* = 3) ± SE

Chlorosis was first visible in the plants treated with calcium chloride (Fig. 2). After 5 days of CaCl_2_ application at 100, 150 and 200 mM, chlorosis symptoms were observed at about 30% of the leaves, and after 14 days all the leaves were clearly chlorotic. After 32 days, chlorosis was visible on the entire plants at all investigated calcium chloride concentrations.

The strongest chlorosis-inducing effect was observed for 150 mM NaCl. The first symptoms for this concentration were visible on the fifth day (ca. 15% of leaves), after 10, 12 and 14 days they could be spotted on about 80% of the leaves and after 24, 27 and 32 days all the leaves were affected. The treatment with 50 mM NaCl induced about 30–50% leaf chlorosis after 10, 12, 14 and 20 days, but after 24, 27 and 32 days about 95% of the leaves were chlorotic. As mentioned previously, chlorosis was earlier visible on the plants treated with CaCl_2_ than on those treated with NaCl.

**Fig. 3:**
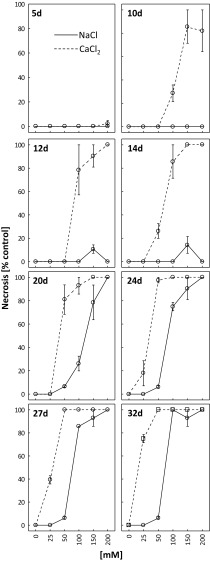
Dynamics of necrosis appearance and its intensity as percent of the control after 5, 10, 12, 14, 20, 24, 27 and 32 days of treating *R. rubiginosa* with NaCl (*solid line*) and CaCl_2_ (*dashed line*) solutions at various concentrations (0, 25, 50, 100, 150, 200 mM). Mean value (*n* = 3) ± SE

Treatment with CaCl_2_ at 100, 150 and 200 mM induced clear leaf tissue necrosis after 10 days of the experiment (Fig. 3). First necroses for 25 mM CaCl_2_ were observed after 24 days. Leaf necrosis rate for the plants treated with this calcium chloride concentration was 40 and 75% after 27 and 32 days, respectively.

No necroses were observed in the plants treated with 25 mM NaCl. Treatment with 50 mM NaCl rarely led to necrosis (10% of leaves). Two hundred millimolars NaCl caused clear necrosis of all leaf tissues after 20 days of the experiment.

**Fig. 4:**
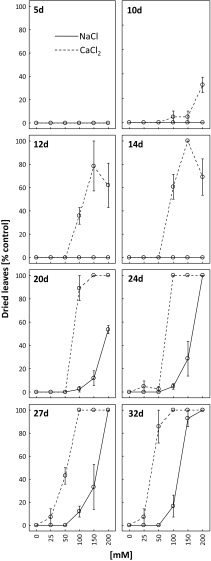
Dynamics of leaf drying and its intensity as percent of the control after 5, 10, 12, 14, 20, 24, 27 and 32 days of treating *R. rubiginosa* with NaCl (*solid line*) and CaCl_2_ (*dashed line*) solutions at various concentrations (0, 25, 50, 100, 150, 200 mM). Mean value (*n* = 3) ± SE

The first symptoms of leaf drying were observed after 10 days of plant treatment with 100, 150 or 200 mM CaCl_2_ (Fig. 4). Complete drying was noticed after 20 days for CaCl_2_ at 100, 150 or 200 mM. After 24, 27 and 32 days of treatment with 200 mM NaCl, drying symptoms could be spotted on all leaves.

The images show the advancement of chlorosis, necrosis and leaf drying in *R. rubiginosa* plants treated with 100 mM NaCl (Fig. 1S) and CaCl_2_ (Fig. 2S). Calcium chloride was more toxic than NaCl, as after 16 days most leaves exposed to CaCl_2_ were completely dry.

### Activity of Photosynthetic Apparatus

**Fig. 5:**
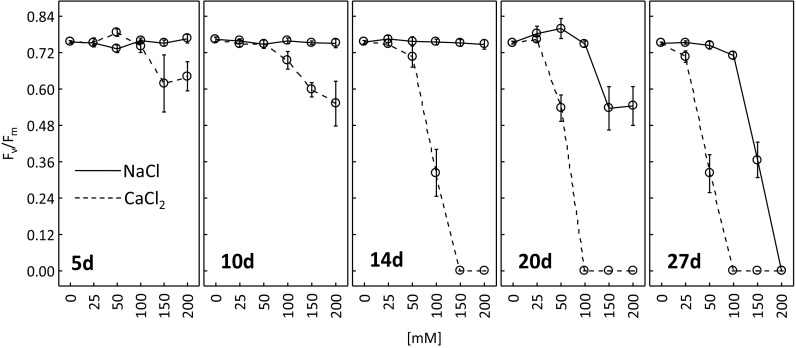
Maximum photochemical efficiency of PSII (*F*_v_/*F*_m_) after 5, 10, 14, 20, and 27 days of treating *R. rubiginosa* with NaCl (*solid line*) and CaCl_2_ (*dashed line*) solutions at various concentrations (0, 25, 50, 100, 150, 200 mM). Mean value (*n* = 5) ± SE

Figure 5 presents alterations in maximum quantum efficiency of photosystem II (*F*_v_/*F*_m_). A considerable decrease in *F*_v_/*F*_m_ was observed after 20 and 27 days in the plants exposed to 150 and 200 mM of sodium chloride. After 5, 10 and 14 days of treating the plants with NaCl solutions of different concentrations, maximum quantum yield of PSII was similar to the control (0 mM).

Calcium chloride was much more detrimental to the photosynthetic apparatus activity. CaCl_2_ at 150 or 200 mM reduced *F*_v_/*F*_m_ after as soon as 5 days (about 83% of control), and after 10 days the decrease was observed at 100 mM (91% of control), 150 mM (78% of control) and 200 mM (71% of control) CaCl_2_. After 14 days, the ratio *F*_v_/*F*_m_ was completely reduced as a result of treatment with 150 and 200 mM CaCl_2_ and the same was observed at 100 mM CaCl_2_ after 20 and 27 days.

**Fig. 6:**
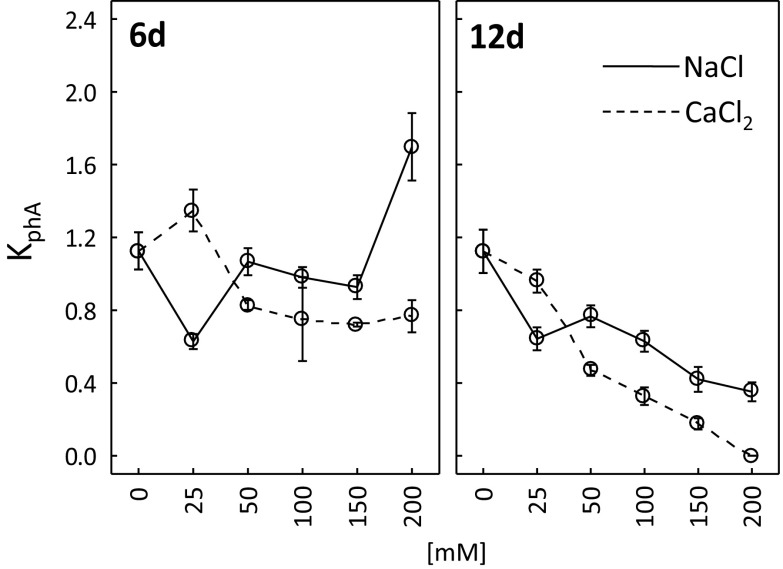
Photosynthetic activity coefficient (*K*_phA_) after 6 and 12 days of treating *R. rubiginosa* with NaCl (*solid line*) and CaCl_2_ (*dashed line*) solutions at various concentrations (0, 25, 50, 100, 150, 200 mM). Mean value (*n* = 5) ± SE

Following 6 days of treatment with 25 mM NaCl, the photosynthetic activity coefficient (*K*_phA_) was clearly lower than in the control plants (0 mM) (Fig. 6). Photosynthetic activity in the plants treated with 50, 100 and 150 mM NaCl was similar to the control but it was higher than that at NaCl concentration of 200 mM. After 12 days, a decrease in photosynthetic activity was perceived for all the treatments, and the lowest K_phA_ was reported for the plants treated with 150 and 200 mM NaCl.

After 6 days of the experiment, photosynthetic activity of the plants exposed to 50, 100, 150 or 200 mM CaCl_2_ was lower than in the control (Fig. 6). However, slight increase in *K*_phA_ was noticed for 25 mM (118% of control) CaCl_2_. *K*_phA_ values were much lower than in the control after 12 days of exposure to 50 (47% of control), 100 (33% of control), 150 (18% of control) and 200 mM (0% of control) CaCl_2_.

### Changes in Leaf Anatomy

**Fig. 7:**
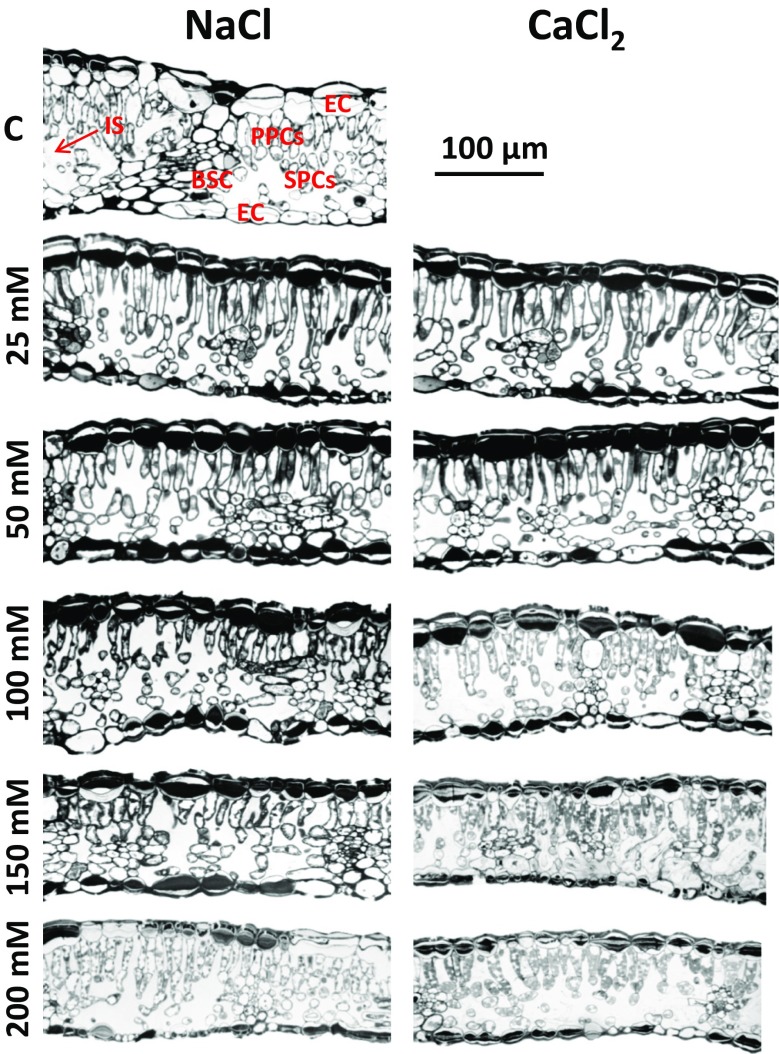
Leaf cross-sections of *R. rubiginosa* after 12 days of treatment with NaCl and CaCl_2_ solutions at various concentrations (0, 25, 50, 100, 150, 200 mM). *EC* — epidermis cell, *PPCs* — palisade parenchyma cells, *SPCs* — spongy mesophyll cells, *BSC* — bundle sheath cell, *IS* — intercellular space

Twelve days of salt treatment resulted in significant changes in leaf anatomy (Fig. 7). Increasing concentration of any salt was accompanied by a decrease in leaf thickness. Considerable changes were visible for the plants treated with 100, 150 and 200 mM of CaCl_2_ and 150 and 200 mM of NaCl. High concentrations of both types of salt caused clear shrinkage of leaf epidermal cells. Moreover, elongation of palisade cells was observed for all NaCl and CaCl_2_ concentrations. Treatment with high concentrations of calcium chloride and sodium chloride (100–200 mM) caused visible deformation of the palisade cells and reduced their density.

### Leaf Water Content and Leaf Dry Weight

**Fig. 8:**
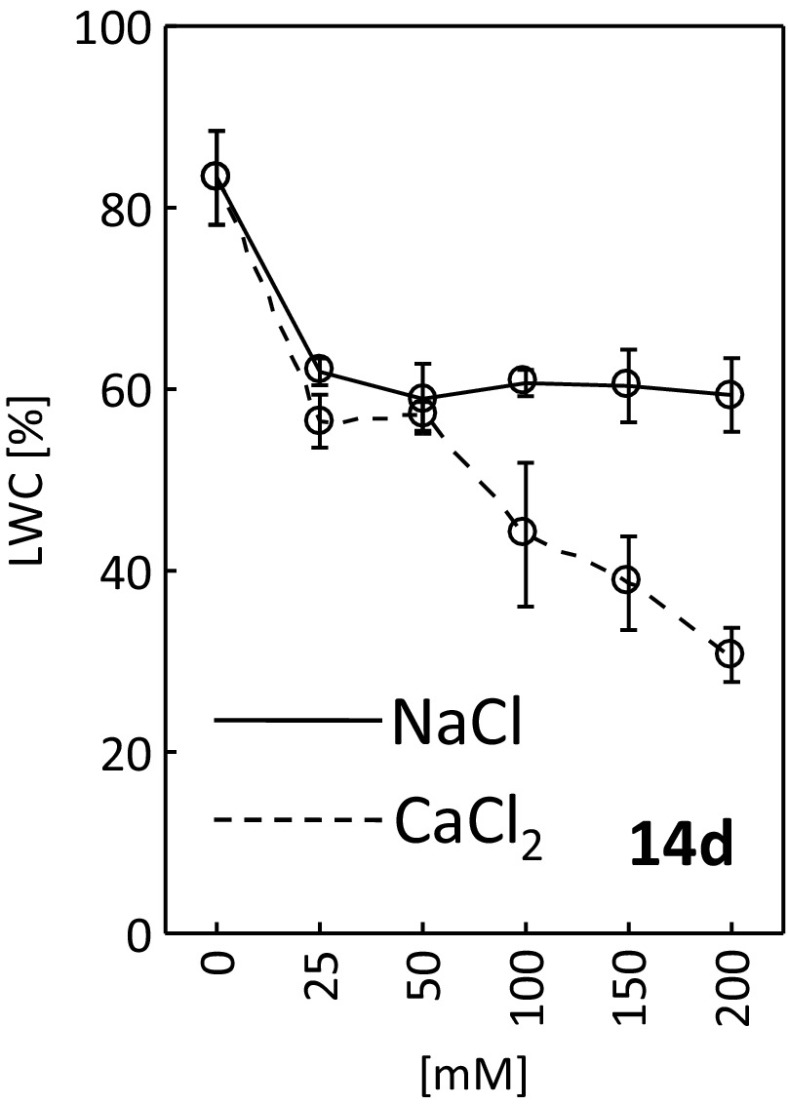
Leaf water content (LWC) after 14 days of treating *R. rubiginosa* with NaCl (*solid line*) and CaCl_2_ (*dashed line*) solutions at various concentrations (0, 25, 50, 100, 150, 200 mM). Mean value (*n* = 7) ± SE

Following 14 days of the experiment, a decrease in leaf water content was perceived in the plants exposed both to NaCl and CaCl_2_ (Fig. 8). LWC for 25, 50, 100, 150 and 200 mM NaCl was about 60% and about 20% less than control (about 85%). In the plants exposed to calcium chloride, a gradual decrease in LWC was seen along with increasing salt concentration. The lowest leaf water content (about 30%) was reported for 200 mM of CaCl_2_.

**Fig. 9:**
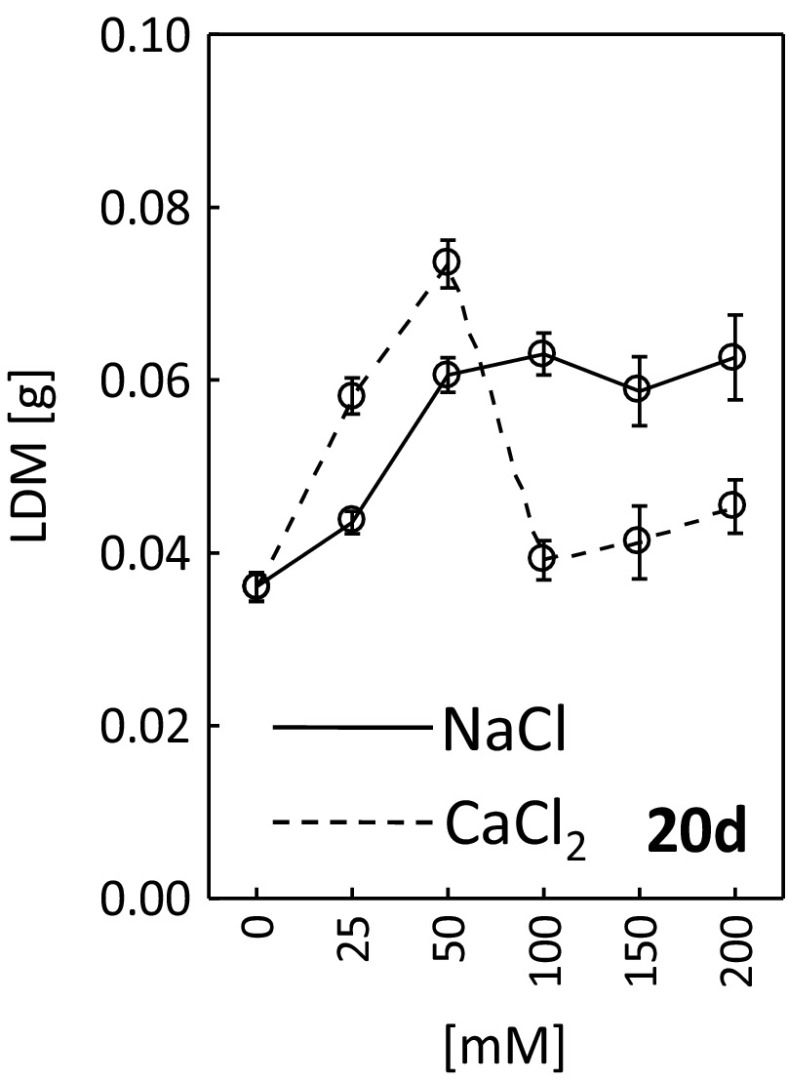
Leaf dry weight (LDW) after 20 days of treating *R. rubiginosa* with NaCl (*solid line*) and CaCl_2_ (*dashed line*) solutions at various concentrations (0, 25, 50, 100, 150, 200 mM). Mean value (*n* = 7) ± SE

All concentrations of NaCl stimulated leaf dry weight (Fig. 9). The same effect was perceived for the treatment with 25 and 50 mM of CaCl_2_, and for the other calcium chloride concentrations (100–200 mM), the dry weight was comparable to the control (0 mM).

## Discussion

The study demonstrated greater accumulation of calcium chloride than sodium chloride in the soil (Table 1). High CaCl_2_ content caused limitations in water availability manifested as physiological drought (Yadav et al. 2011) that finally led to a decrease in leaf water content (Fig. 8) and fast leaf drying (Fig. 4, Fig. 2S). Sohan et al. (1999) and Romero-Aranda et al. (2001) demonstrated that increased salinity in the root zone resulted in lower leaf water content and led to the disruption of many important plant physiological processes. A similar decrease in leaf water content triggered by salt stress was observed in other studies (Ghoulam et al. 2002; Katerji et al. 1997; Kerepesi and Galiba 2000; Rivero et al. 2013; Turkan et al. 2013).

*R. rubiginosa* was more sensitive to the salinity induced by calcium chloride than by sodium chloride. Plant response to salinity was variable and depended on the salt concentration. In general, toxic effects were exacerbated by increasing salt concentration and exposure time. Our results have confirmed a well-known fact that chloride ions in the form of CaCl_2_ are more toxic to plants than other salts, such as NaCl or KCl (Cram 1973; Grattan and Grieve 1999; Kafkafi et al. 1992; Pessarakli 1991).

The effects of both types of salt on *R. rubiginosa* condition were manifested in the form of leaf chlorosis and necrosis (Figs. 2 and 3, Fig. 1S, Fig. 2S). Both changes were first perceived in the plants treated with calcium chloride, and this further confirmed greater toxicity of this salt towards *R. rubiginosa*. Chlorosis and necrosis triggered by substrate salinity were also described by other authors (Chan et al. 2011; Koffler et al. 2015; Paludan-Muller et al. 2002; Slabu et al. 2009). Wahome et al. (2001) reported higher tolerance of *R. rubiginosa* to NaCl, as compared with *Rosa chinensis* that was manifested by more pronounced leaf necroses in the latter species. It should be pointed here that the treatment with 150 mM NaCl resulted in higher extent of leaf chlorosis compared to 200 mM (5, 10, 12, 14 and 20 days of treatment) (Fig. 2). We propose explanation that higher salt concentration may induce a more effective defence mechanisms than at lower salt concentration (Khan et al. 2000; Walia et al. 2007).

It is well-known that plants dynamically acclimate their photosynthetic system to environmental conditions (Repkova et al. 2009; Schurr et al. 2006). CaCl_2_ was more detrimental to the performance of the photosynthetic apparatus (Fig. 5) and photosynthetic activity (Fig. 6) than NaCl. The studies of other authors also demonstrated a negative impact of salt stress on plant photosynthetic activity (Agastian et al. 2000; Dionisio-Sese and Tobita 1998; Flexas et al. 2004; Kalaji et al. 2016; Koyro 2006; Tang et al. 2015). This may be due to, amongst others, lowered membrane permeability to CO_2_ (Iyengar and Reddy 1996), reduced nitrogen absorption from the soil (Fisarakis et al. 2001), stomatal closure (Parida et al. 2004) or decreased enzymatic activity (Iyengar and Reddy 1996). Salt stress was also reported to stimulate photosynthesis and plant biomass growth (Gu et al. 2016; Kurban et al. 1999; Parida et al. 2004; Redondo-Gómez et al. 2007). Our experiment demonstrated a stimulating effect of both low (25, 50 mM) and high (100, 150, 200 mM) concentrations of NaCl and low concentrations of CaCl_2_ (25, 50 mM) on dry weight of *R. rubiginosa* (Fig. 9). Khan et al. (2000) and Walia et al. (2007) claimed that the stimulating effect of salinity on plant dry weight might be due to increased concentrations of plant growth regulators (e.g. jasmonic acid) that may indirectly activate the genes (Rubisco, Rubisco activase) related to the photosynthetic activity.

Treatment with CaCl_2_ caused more visible deformation of palisade cells, reduced their density and considerably reduced leaf thickness (Fig. 7). However, it is advisable to confirm the changes in leaf anatomy of *R. rubiginosa* by precise measurements of epidermal thickness, mesophyll thickness, palisade cell length, palisade cell diameter, spongy cell diameter, stomatal density and intercellular spaces in future studies. Salt stress-induced changes in the anatomy of *R. rubiginosa* leaves were concurrent with the results of other studies (Garcia‐Abellan et al. 2015; Longstreth and Nobel 1979; Parida et al. 2004; Romero-Aranda et al. 2001; Yadav et al. 2011). These reports discussed the role of some leaf parameters and structures in the photosynthesis under salt stress.

Our study showed that *R. rubiginosa* has higher tolerance to salt stress induced by NaCl than by CaCl_2_. Visual effects of plant response to salt stress were variable and depended on the salt concentrations. High concentrations of NaCl and CaCl_2_ (100–200 mM) induced more intense chlorosis, necrosis and leaf drying than low concentrations of these salts (25–50 mM). Summing up, we suggest that *R. rubiginosa* may be a natural indicator of urban soil salinity, particularly in the soils lining the communication routes where chemical agents to reduce road slippery are used.

## Electronic Supplementary Material

Below is the link to the electronic supplementary material.

ESM 1 Figure 1S. Images showing the dynamics of chlorosis, necrosis and leaf drying after 3, 10, 16, 22, 27 and 32 days of the experiment for the same *R. rubiginosa* plant treated with 100 mM NaCl. (PPTX 495 kb)

ESM 2 Figure 2S. Images showing the dynamics of chlorosis, necrosis and leaf drying after 3, 10, 16, 22, 27 and 32 days of the experiment for the same *R. rubiginosa* plant treated with 100 mM CaCl_2_. (PPTX 311 kb)
