# Supplementary figures and images for: Physiological Responses of Rosa rubiginosa to Saline Environment
Source: Water Air Soil Pollut. 2017 Jan 28;228(2):81. doi: 10.1007/s11270-017-3263-2 (PMC5274639; doi:10.1007/s11270-017-3263-2)

## Slide 1
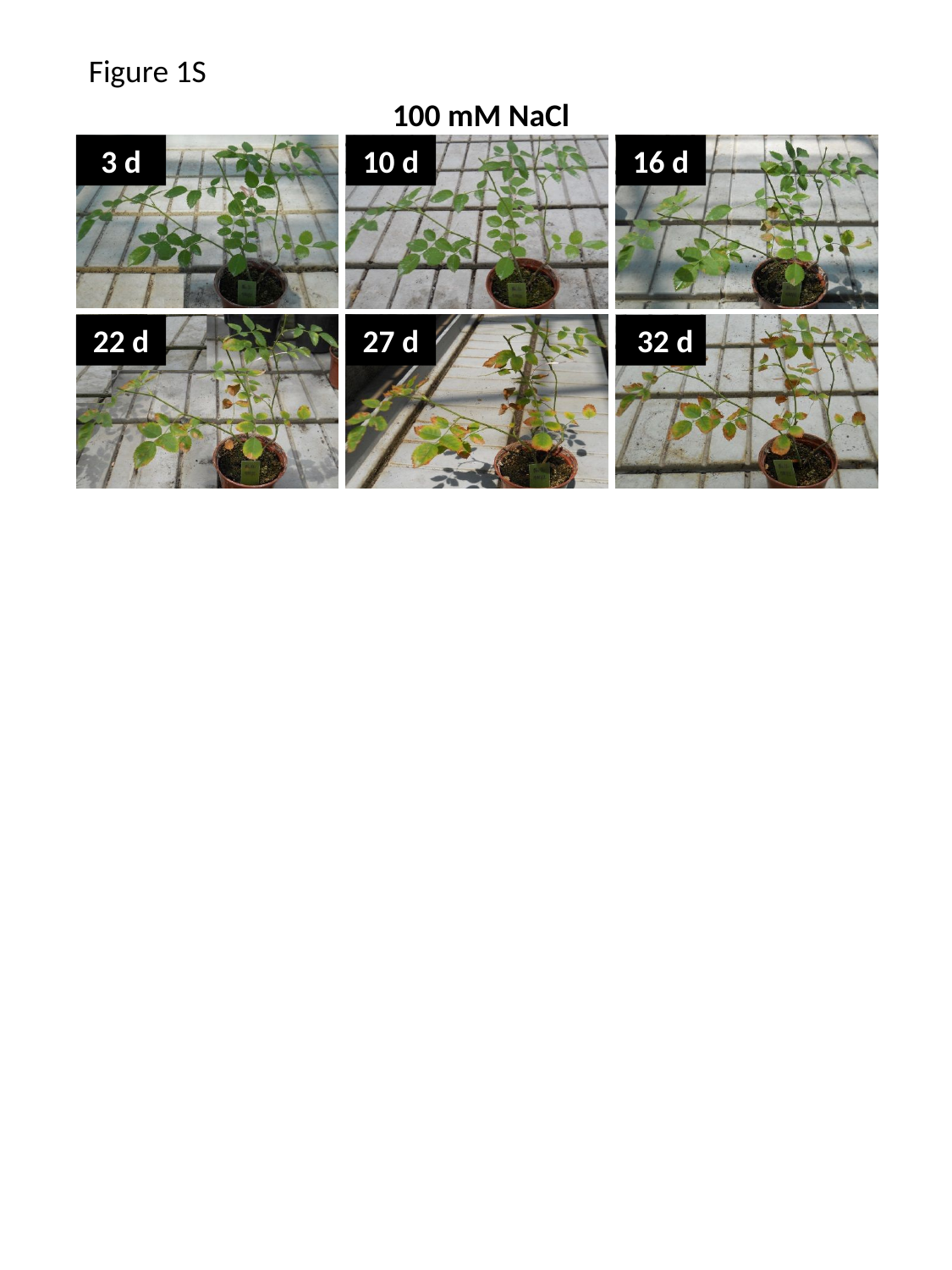

Figure 1S
100 mM NaCl
3 d
10 d
16 d
22 d
27 d
32 d

Supplement: Supplementary file 1 — Figure 1S. Images showing the dynamics of chlorosis, necrosis and leaf drying after 3, 10, 16, 22, 27 and 32 days of the experiment for the same R. rubiginosa plant treated with 100 mM NaCl. (PPTX 495 kb) [file 11270_2017_3263_MOESM1_ESM.pptx]

## Slide 1
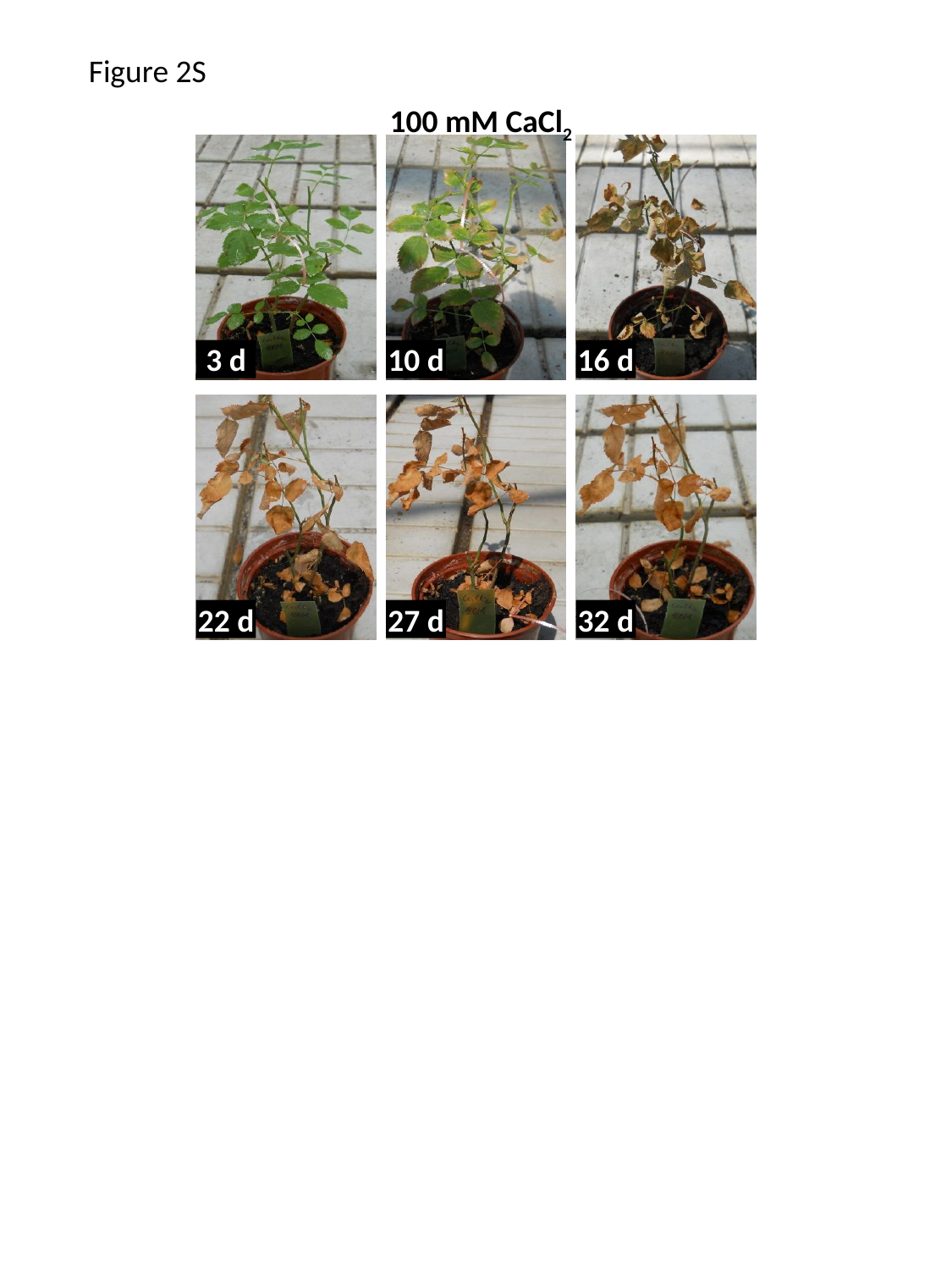

Figure 2S
100 mM CaCl2
3 d
10 d
16 d
22 d
27 d
32 d

Supplement: Supplementary file 2 — Figure 2S. Images showing the dynamics of chlorosis, necrosis and leaf drying after 3, 10, 16, 22, 27 and 32 days of the experiment for the same R. rubiginosa plant treated with 100 mM CaCl2. (PPTX 311 kb) [file 11270_2017_3263_MOESM2_ESM.pptx]
